# Stereoselective 15α-hydroxylation of androsta-1,4-diene-3,17-dione by *Gibberella* sp. for efficient production of 15α-OH-ADD and 15α-OH-AD

**DOI:** 10.1039/d5ra08374e

**Published:** 2026-01-22

**Authors:** Ran He, Ming Song, Ruicheng Fu, Yueying Jin, Shi Li, Fuju Wang, Guogang Zhang, Yongbo Song, Weizhuo Xu

**Affiliations:** a School of Traditional Chinese Materia Medica, Shenyang Pharmaceutical University Shenyang 110016 China; b School of Life Sciences and Biopharmaceuticals, Shenyang Pharmaceutical University Shenyang 110016 China songyongbo@syphu.edu.cn +86-024-43520900 +86-024-43520900; c School of Functional Food and Wine, Shenyang Pharmaceutical University Shenyang 110016 China weizhuo.xu@syphu.edu.cn +86-024-43520301 +86-024-43520301; d Surveying and Mapping Department, Shenyang Urban Construction University 110167 China; e Beijing Global Biologicals Inc. Beijing 100192 China

## Abstract

Androsta-1,4-diene-3,17-dione (ADD) and its hydroxylated derivatives are important steroidal drug intermediates, playing a crucial role in the pharmaceutical industry. This study evaluated the whole-cell biotransformation of ADD by *Gibberella* sp. CICC 2498 and *Absidia* sp. CICC 41050. While *Absidia* sp. showed low efficiency, *Gibberella* sp. selectively produced two major metabolites, identified as 15α-OH-ADD and 15α-OH-AD *via* HPLC, MS, and NMR. Through orthogonal design and single-factor experiments, the transformation rate of ADD to 15α-OH-ADD was enhanced from 30.35% to 61.74% under optimal conditions: 1 g L^−1^ ADD, 12% inoculum, 70 mL medium volume, 120 h incubation, and 1% (v/v) methanol as cosolvent. These results highlight *Gibberella* sp. as a promising biocatalyst for regio- and stereoselective steroid hydroxylation, providing an efficient microbial strategy for high-value steroidal intermediates in pharmaceutical manufacturing.

## Abstract 2

Biocatalytic hydroxylation of steroids by *Gibberella* sp. in steroid intermediate synthesis. Products identification and biotransformation optimization. Efficient 15α-OH-ADD production and industrial potential by *Gibberella* sp.

## Graphical abstract

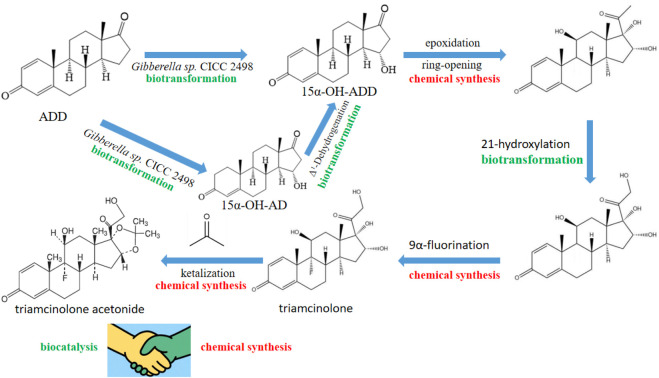

## 1. Introduction

Steroids are widely distributed among microorganisms, plants, and animals. They exhibit diverse physiological functions depending on their structural variations, such as the position and stereochemistry of double bonds on the steroidal nucleus, as well as the type, position, and number of substituents.^1,2^ Steroids possess extensive biological activities, with naturally occurring steroids playing essential roles in metabolism, signal transduction, immunity, reproduction, and mineralocorticoid regulation.^3–5^ In the pharmaceutical field, over 400 steroidal drugs have been approved globally, with market demand second only to antibiotics and projected to grow further.^6,7^

Conventionally, steroidal compounds are produced through multi-step chemical synthesis starting from diosgenin derived from plant sources such as *Dioscorea* species.^8^ Alternatively, steroidal intermediates or non-steroidal precursors are extracted directly from animal or plant tissues and subsequently converted through complex chemical synthesis.^9^ However, these traditional processes suffer from multiple drawbacks, including complicated synthetic routes, low yields, high energy consumption, and severe environmental pollution, rendering them unsustainable in meeting the increasing demands of the steroid industry.^10,11^ Considering the growing global demand for steroid hormones and the rapid depletion of traditional plant-derived steroid resources, microbial biotransformation has emerged as a promising and sustainable alternative.^12,13^ Recent research efforts have increasingly focused on microbial biotransformation methods for steroid synthesis, highlighting advantages such as operational simplicity, mild reaction conditions, environmental friendliness, improved stereoselectivity, and higher economic viability, thus providing an important direction for future development in the steroid industry.^14,15^ Microbial biotransformation of steroids encompasses a variety of reaction types, including hydroxylation, dehydrogenation, steroidal nucleus degradation, steroidal side-chain cleavage, oxidation of hydroxyl groups to ketones, epoxidation, aromatization of ring A, hydrolysis, esterification, isomerization, resolution of racemic mixtures, and amination.^16,17^ Among these reactions, site-specific hydroxylation significantly influences the physiological activity of steroids.^18^ Microorganisms possess the capability to hydroxylate steroids at multiple sites on the steroid nucleus, including inert or unactivated C–H bonds, which are challenging to modify using conventional chemical methods.^13^ Additionally, microbial hydroxylation reactions typically yield products with high regioselectivity and stereoselectivity, selectively generating either α- or β-configured hydroxyl groups, thus effectively addressing issues related to poor selectivity and excessive byproduct formation inherent in chemical synthesis.^18^ For instance, using testosterone as a substrate, *Absidia glauca* AM177 efficiently catalyzes hydroxylation to produce 7α-OH-AD and 7β-OH-AD.^19^ Similarly, employing androsta-1,4-diene-3,17-dione (ADD) as the substrate, *Gongronella butleri* can specifically yield 6β-OH-ADD, 7β-OH-ADD, and 14α-OH-ADD.^20^ Notably, 7β-hydroxylated steroids and their derivatives demonstrate significant neuroprotective and anti-inflammatory activities, rendering them promising candidates for therapeutic applications in chronic neuronal injury.^21^ Moreover, the introduction of hydroxyl groups at the 9α-position facilitates subsequent halogenation reactions, significantly enhancing the therapeutic potency of glucocorticoid drugs such as dexamethasone and mometasone furoate.^22^ Additionally, the 11α-hydroxylation of steroids is critical in synthesizing intermediates for eplerenone, a drug clinically utilized for treating cardiovascular diseases such as hypertension, heart disease, and proteinuria.^23^ 11β-hydroxylation significantly contributes to the anti-inflammatory potency of steroids, enabling the synthesis of prominent glucocorticoids such as hydrocortisone and prednisolone.^24^ Furthermore, 14α-hydroxylated steroids and their derivatives are recognized as potent aromatase inhibitors possessing contraceptive and anticancer activities and serve as precursors for highly effective anti-gonadotropic drugs such as promegestone.^25^ The 15α-hydroxylation of steroids represents a crucial reaction pathway in the synthesis of pregnane derivatives. Specifically, 15α-hydroxysteroids serve as essential intermediates in the production of contraceptive agents, notably progesterone, one of the most widely used contraceptive steroids clinically.^26^ Qiao *et al.* reported that *Colletotrichum lini* AS3.4486 can biotransform androsta-1,4-diene-3,17-dione (ADD) into multiple hydroxylated metabolites, including 15α-OH-ADD, 11,15-diOH-ADD, and 15,17β-dihydroxyandrost-1,4-dien-3-one (15-OH-BD). Intriguingly, a detailed kinetic analysis of the hydroxylation process revealed regioselective hydroxylation occurring specifically at the C-15 position, followed by sequential hydroxylations leading to the formation of 11,15-diOH-ADD and 15-OH-BD. Enzyme inhibition assays confirmed that hydroxylations at the C-11 and C-15 positions were catalyzed by distinct hydroxylase enzymes.^27^ The synthesis of some steroidal antitumor drugs (such as aromatase inhibitors) requires 15 hydroxyl groups as active groups. 15α-OH-ADD can produce antitumor drugs with the effect of inhibiting estrogen synthesis through structural modification for breast cancer treatment.^28^ Although filamentous fungi, such as *Gibberella* sp. and *Absidia coerulea*, are widely recognized for their remarkable ability to perform regio- and stereospecific hydroxylation of androstane- and pregnane-type steroids,^29,30^ their potential for biotransforming androsta-1,4-diene-3,17-dione (ADD) into high-value steroid derivatives has not yet been fully explored.

**Fig. 1:**
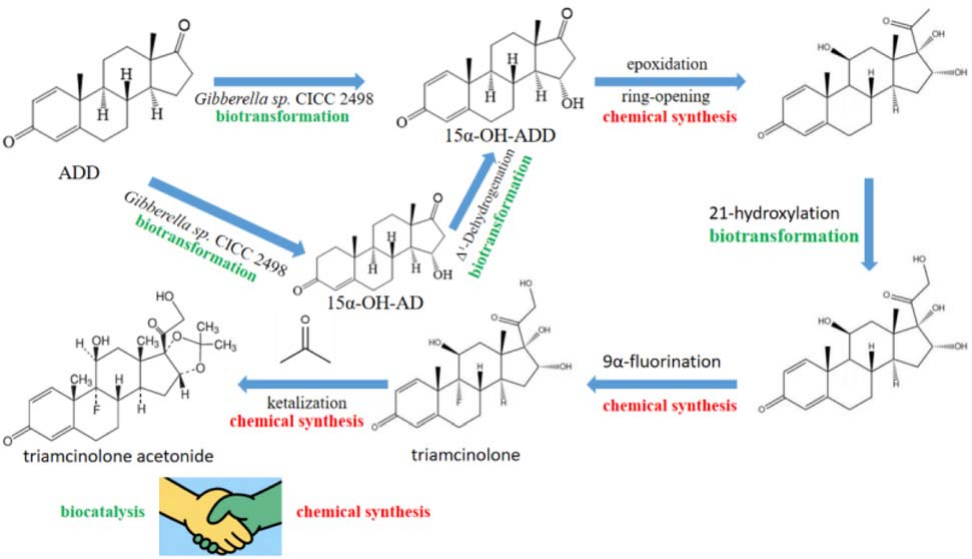
15α-OH-ADD and 15α-OH-AD obtained by *Gibberella* sp. CICC 2498. The chemo-biosynthetic strategy proves to be a green and highly efficient approach. Both 15α-OH-ADD and 15α-OH-AD serve as key advanced intermediates in the synthesis of 16α-hydroxylated corticosteroids.

In the present study, we demonstrated that two fungal strains, *Gibberella* sp. CICC 2498 exhibited notable steroid biotransformation activities, effectively converting ADD into valuable steroid intermediates (15α-OH-ADD and 15α-OH-AD) (Fig. 1). 15α-OH-ADD is a crucial core intermediate for the synthesis of highly potent glucocorticoids such as triamcinolone and triamcinolone acetonide. Our results highlight these microorganisms as promising biocatalysts for industrial steroid transformations due to their excellent regio- and stereoselectivity, biocatalytic stability, and productivity. These findings significantly expand the potential industrial applications of *Gibberella* sp., underscoring its suitability for developing more sustainable, cost-effective, and environmentally friendly steroid manufacturing processes.

## 2. Materials and methods

### 2.1. Chemicals

Androst-1,4-diene-3,17-dione (ADD) was obtained from Hubei Gongtong Pharmaceutical Co., Ltd (Hubei, China). Methanol and acetonitrile were purchased from Concord Technology Co., Ltd (Tianjin, China). Yeast extract was purchased from HopeBio Co., Ltd (Qingdao, China). All other chemical reagents were procured from Yuwang Chemical Co., Ltd (Shenyang, China).

### 2.2. Microorganisms and cultivation

*Gibberella* sp. CICC 2498 and *Absidia coerulea* CICC 41050 were acquired from the China Center of Industrial Culture Collection (CICC). Potato dextrose agar (PDA) was pruchased from Hopebio (Qingdao, China). The seed culture medium (g L^−1^) contained potato starch (45 g), yeast extract (3 g), corn steep liquor (10 g), CaCO_3_ (3 g), MgSO_4_ (0.5 g), and FeSO_4_ (0.05 g).^31^ The transformation medium (g L^−1^) was comprised of sucrose (30 g), yeast extract (10 g), corn steep liquor (10 g), K_2_HPO_4_ (2 g), KH_2_PO_4_ (1.6 g), MgSO_4_ (0.5 g), FeSO_4_ (0.05 g), and adjusted to pH 6.5.

The fungi were routinely maintained on PDA slants. To obtain the first generation mycelium, the spore suspension from a one-week-old slant was inoculated into 50 mL of seed culture medium in 250 mL Erlenmeyer flasks and incubated aerobically at 28 °C on a rotary shaker (200 rpm) for 48 hours. Subsequently, 5 mL of seed culture was inoculated into 50 mL of transformation medium in a 250 mL flask and cultured under the same conditions for 5 days. Substrate control groups were prepared without inoculating fungi, and strain controls were prepared without adding substrate, while maintaining the same conditions.

### 2.3. Sample preparation

After 5 days of culture, the broth was centrifuged at 3000 rpm for 10 minutes to separate the mycelia from the transformation solution. Both the mycelia and transformation solution were extracted three times with an equal volume of ethyl acetate. The combined extracts were evaporated under reduced pressure using a rotary evaporator, then redissolved in 5 mL methanol. An appropriate amount of anhydrous magnesium sulfate was added to dry the sample, which was then used for analysis.

### 2.4. Thin layer chromatography (TLC)

The concentrated extract was analyzed by TLC using silica gel 60 F254 plates (Merck)^29^ and a solvent mixture of CHCl_3_-acetone (1 : 1, v/v) as the mobile phase.^32,33^ The metabolites were visualized by spraying the plates with a mixture of H_2_SO_4_/ethanol (1 : 9, v/v), followed by observation under UV light at 254 nm.

### 2.5. HPLC detection

A 0.1 mL aliquot of the conversion product containing ADD was diluted five times with methanol and filtered using a 0.45 µm organic membrane. HPLC analysis was performed on a WondaSil C18 Superb column (5 µm, 4.6 mm × 250 mm, Shimadzu, Japan) with a methanol/water mixture (60 : 40, v/v) as the mobile phase at 25 °C and UV detection at 242 nm. Flow rate was 0.7 mL min^−1^, and the injection volume was 10 µL.^34,35^

### 2.6. Isolation and identification of major metabolites

Semi-preparative HPLC was used to isolate the target metabolites, with separation performed on a SinoChrom ODS-BP column (5 µm, 10 mm × 250 mm, Elite, China) using an acetonitrile/water mixture (35 : 65, v/v) as the mobile phase at 36 °C, and UV detection at 242 nm. The flow rate was 3.8 mL min^−1^, and the injection volume was 100 µL. Purified metabolites were identified using ESI-MS and NMR. ^1^H and ^13^C NMR spectra were recorded using a Brüker AVANCE III 400 NMR instrument (Bruker Biospin AG, Switzerland) in CDCl_3_ or DMSO-*d*_6_, with tetramethylsilane (TMS) as the internal standard. Mass spectrometry was conducted in ESI mode on an Agilent 1200 LC-MS system (Agilent, USA).

HPLC was used to establish a standard curve plotting the concentration of 15α-OH-ADD (abscissa) against the peak area (ordinate). The transformation rate was calculated using the previously established methodology in our laboratory.^31^

### 2.7. Optimization of ADD transformation to 15α-OH-ADD by *Gibberella* sp. CICC 2498

To optimize the biotransformation of ADD to 15α-OH-ADD by *Gibberella* sp. CICC 2498, various organic solvents were evaluated for their ability to enhance substrate solubility and biotransformation efficiency. Specifically, 50 mg of ADD was separately dissolved in 1 mL of selected organic solvents prior to introduction into the biotransformation system. Based on the selection of the optimal organic cosolvent, single-factor experiments were carried out to investigate the influence of individual medium components on the biotransformation efficiency. High-performance liquid chromatography (HPLC) was employed to quantify and analyze the biotransformation results.

Building upon the optimized cosolvent system, single-factor experiments were initially conducted to assess the effects of medium composition (carbon/nitrogen sources) and pH on transformation efficiency. These findings guided the implementation of an orthogonal experimental design. Subsequently, based on the optimized cosolvent and medium formulation, additional single-factor experiments were conducted followed by an orthogonal array design to systematically evaluate critical process parameters, including inoculum size, medium volume, fermentation time, and substrate concentration.

### 2.8. Statistical analysis

SPSS 20.0 software was used to conduct *T*-test on the data to determine the statistical difference, *p* < 0.05 was significant, *p* < 0.01 was extremely significant.

## 3. Results and discussion

### 3.1. TLC analysis of ADD biotransformation by *Absidia coerulea* CICC 41050 and *Gibberella* sp. CICC 2498

**Fig. 2:**
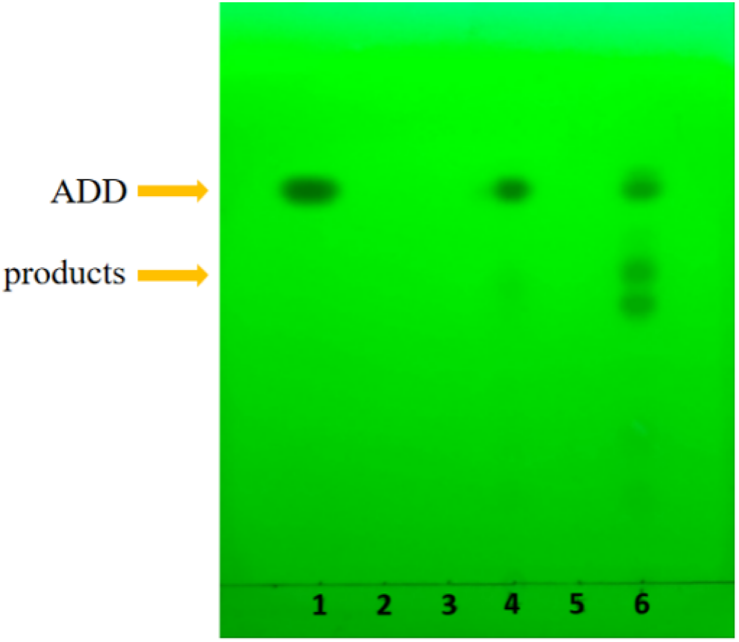
TLC analysis of the microbial transformation of androsta-1,4-diene-3,17-dione (ADD). 1: Substrate control (ADD, 1 g L^−1^; methanol, 1 mL). 2: Cosolvent control (methanol, 1 mL) only. 3: *Absidia coerulea* CICC 41050 (10% inoculum) only. 4: *Absidia coerulea* CICC 41050 + ADD (10% inoculum; ADD, 1 g L^−1^; methanol, 1 mL). 5: *Gibberella* sp. CICC 2498 (10% inoculum) only. 6: *Gibberella* sp. CICC 2498 + ADD (10% inoculum; ADD, 1 g L^−1^; methanol, 1 mL). All groups were incubated at 220 rpm, 28 °C, in 50 mL of medium for 120 hours.

The preliminary biotransformation of androsta-1,4-diene-3,17-dione (ADD) by *Absidia coerulea* CICC 41050 and *Gibberella* sp. CICC 2498 was evaluated using thin-layer chromatography (TLC), and the results are presented in Fig. 2. As indicated in Fig. 2, additional spots aside from the substrate ADD appeared in the TLC profiles for both fungal strains, demonstrating their capacity to convert ADD into steroid derivatives. However, notable differences were observed between the two strains. Specifically, the biotransformation products generated by *Absidia coerulea* CICC 41050 displayed faint spots, suggesting limited biotransformation activity. In contrast, *Gibberella* sp. CICC 2498 produced clearly visible and well-resolved product spots, indicating higher specificity and efficiency in transforming ADD to steroidal intermediates. The results from the TLC analysis preliminarily indicated that *Gibberella* sp. CICC 2498 exhibited greater potential as a biocatalyst for ADD transformation compared to *Absidia coerulea* CICC 41050. TLC provides a rapid, efficient, and highly adaptable technique for initial evaluation of biotransformation processes, especially when handling crude extracts without prior purification.^36^ Its widespread use in preliminary steroid analysis stems from several advantages, including simple sample preparation, short analytical duration, high sensitivity, minimal equipment requirements, and the capability of simultaneously processing multiple samples.^32^ These findings provide an essential foundation for further optimization and more detailed analytical characterization, such as HPLC and mass spectrometry (MS), to accurately identify and quantify specific steroid transformation products.

### 3.2. HPLC analysis of ADD biotransformation by *Absidia coerulea* CICC 41050 and *Gibberella* sp. CICC 2498

**Fig. 3:**
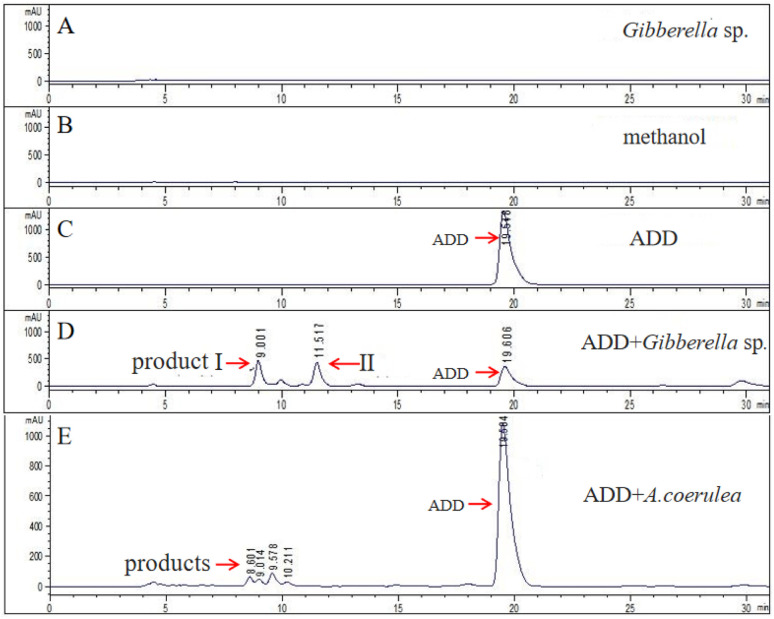
HPLC analysis of the transformation of ADD by *Gibberella* sp. CICC 2498 and *A. coerulea* CICC 41050. Peaks labeled as I and II correspond to the two main transformation products of ADD generated by *Gibberella* sp. CICC 2498. (A) Microbial control group; (B) cosolvent control group; (C) substrate control group. (D) *Gibberella* sp. transformation group. (E) *A. coerulea* transformation group.

The biotransformation of androsta-1,4-diene-3,17-dione (ADD) by *Gibberella* sp. CICC 2498 was further investigated by HPLC analysis, as shown in Fig. 3. Fig. 3A–C represent the control groups, including the microbial control (*Gibberella* sp. CICC 2498 without ADD, Fig. 3A), the cosolvent control (methanol without ADD and microorganism, Fig. 3B), and the substrate control (ADD without microorganism, Fig. 3C), respectively. The HPLC profile obtained from the biotransformation of ADD by *Gibberella* sp. CICC 2498 is presented in Fig. 3D. Compared with the substrate control (Fig. 3C), the peak corresponding to ADD significantly diminished in the biotransformation group (Fig. 3D), clearly demonstrating that ADD underwent microbial transformation by *Gibberella* sp. CICC 2498 rather than spontaneously degrading within the medium. Moreover, the additional peaks observed exclusively in the biotransformation group (Fig. 3D), which were absent in both the microbial control (Fig. 3A) and solvent control (Fig. 3B), confirmed that these newly formed peaks represented the specific biotransformation products of ADD, rather than metabolites produced through microbial growth alone. The HPLC results indicate that *Gibberella* sp. CICC 2498 predominantly converted ADD into a limited number of products with distinct and well-resolved peaks, thereby facilitating subsequent isolation and purification processes. Two major biotransformation products were clearly detected and designated as product I and product II, which displayed retention times of 9.001 min and 11.517 min, respectively. Considering the significant abundance and distinct retention times of products I and II, further work will focus on the isolation, purification, and structural elucidation of these products. Such studies aim to thoroughly characterize their chemical structures and evaluate their potential pharmaceutical or industrial applications. As shown in Fig. 3E, the transformation of ADD by *Absidia coerulea* CICC 41050 resulted in a low transformation rate, with multiple distinct product peaks. These findings were consistent with the TLC results, where *Absidia coerulea* CICC 41050 exhibited weak transformation efficiency (Fig. 2).

### 3.3. Isolation, purification, and structural identification of products I and II

**Fig. 4:**
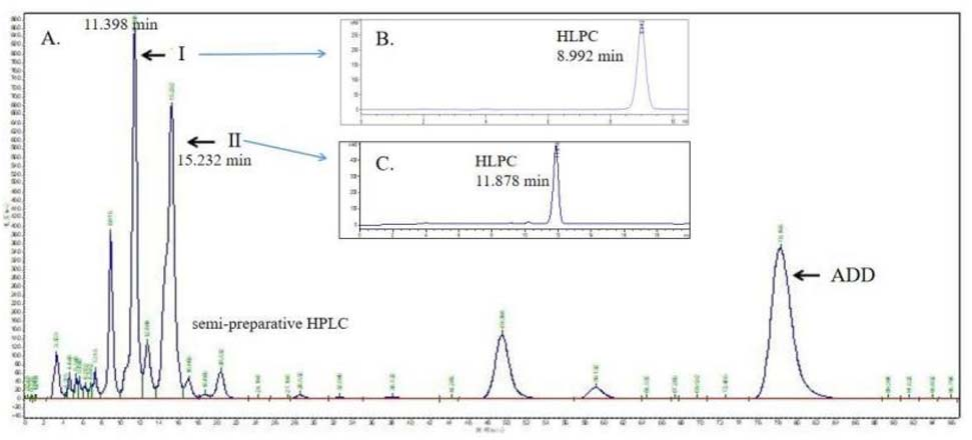
Semi-preparative HPLC separation of products. (A) Semi-preparative HPLC separation of products I and II from the fermentation broth after incubation of *Gibberella* sp. CICC 2498 with ADD (1 g L^−1^, 220 rpm, 28 °C, 120 h). (B) HPLC identification of product I. (C) HPLC identification of product II.

The biotransformation products of ADD by *Gibberella* sp. CICC 2498 were isolated and purified using semi-preparative HPLC, as depicted in Fig. 4A. The semi-preparative chromatogram (Fig. 4A) revealed two prominent peaks with retention times of 11.398 min (product I) and 15.232 min (product II), respectively. Subsequently, the purified fractions corresponding to products I and II were re-analyzed using analytical HPLC to confirm their purity and identity (Fig. 4B and C). Analytical HPLC analysis indicated that product I exhibited a retention time of 8.992 min (Fig. 4B), whereas product II displayed a retention time of 11.878 min (Fig. 4C). When compared with the retention times observed in the original biotransformation mixture (Fig. 3D), these results conclusively confirmed that the isolated and purified compounds corresponded to the main ADD biotransformation products formed by *Gibberella* sp. CICC 2498.

Further structural identification, including NMR spectroscopy, mass spectrometry (MS), and other relevant analytical methods, will be conducted to comprehensively characterize the chemical structures of products I and II. The structural elucidation of these purified metabolites will provide insights into their potential biological activities and pharmaceutical relevance, thus paving the way for potential therapeutic applications or further industrial development. The collected eluents from semi-preparative HPLC were concentrated to dryness under reduced pressure using a rotary evaporator, and subsequently redissolved in deuterated chloroform (CDCl_3_) for structural analyses *via* MS and NMR. According to the obtained MS (ESI) spectrum of product I ([M + H]^+^ at *m*/*z* 301.17, Fig. S1A), the molecular weight of product I was deduced to be 300, corresponding to the molecular formula C_19_H_24_O_3_. Compared to the molecular weight of the substrate ADD (C_19_H_24_O_2_, MW 284), product I exhibited an increase corresponding precisely to one additional oxygen atom, indicating the introduction of a hydroxyl group. Similarly, based on the MS (ESI) spectrum of product II ([M + H]^+^ at *m*/*z* 303.20, Fig. S1B), the molecular weight of product II was calculated to be 302, corresponding to the molecular formula C_19_H_26_O_3_. Compared to ADD, the increase in molecular weight by 18 units suggested the addition of one oxygen atom accompanied by the reduction of one double bond within the steroid structure. The exact positions of the introduced hydroxyl groups and structural modifications in products I and II were further elucidated by comprehensive ^13^C NMR and ^1^H NMR spectroscopic analyses(Fig. S2 and S3), summarized in Table 1. These analyses allowed precise identification of the structural changes resulting from the biotransformation of ADD by *Gibberella* sp. CICC 2498, enabling a detailed interpretation of the regio- and stereospecific enzymatic modifications involved.

**Table 1:** NMR spectroscopic data (^13^C NMR:151 MHz, CDCl_3_; ^1^H NMR: 600 MHz, CDCl_3_) for product I and product II

Position	Product I		Product II	
	*δ*_C_	*δ*_H_	*δ*_C_	*δ*_H_
1	155.33	7.04	35.87	
2	127.97	6.25	34.04	
3	186.36		199.43	
4	124.08	6.09	124.14	5.75
5	168.40		170.26	
6	33.64		32.74	
7	32.78		31.44	
8	35.49		35.59	
9	52.49		53.84	
10	43.53		38.80	
11	22.19		20.39	
12	31.37		32.10	
13	46.26		50.68	
14	57.22		57.58	
15	70.32	4.39–4.45	70.53	4.41–4.45
16	50.82		46.42	
17	215.54		215.87	
18	15.57	0.98	15.49	0.96
19	18.93	1.27	17.65	1.23

For product I, ^13^C NMR (151 MHz, CDCl_3_): *δ* 215.54, 186.36, 168.40, 155.33, 127.97, 124.08, 70.32, 57.22, 52.49, 50.82, 46.26, 43.53, 35.49, 33.64, 32.78, 31.37, 22.19, 18.93, 15.57. ^1^H NMR (600 MHz, CDCl_3_): *δ* 7.04 (1H, d, *J* = 10.1 Hz, H-1), 6.25 (1H, d, *J* = 12.1 Hz, H-2), 6.09 (1H, s, H-4), 4.45–4.39 (1H, m, H-15), 1.27 (3H, s, H-19), 0.98 (3H, s, H-18).

For product II: ^13^C NMR (151 MHz, CDCl_3_): *δ* 215.87, 199.43, 170.26, 124.14, 70.53, 57.58, 53.84, 50.68, 46.42, 38.80, 35.87, 35.59, 34.04, 32.74, 32.10, 31.44, 20.39, 17.65, 15.49. ^1^H NMR (600 MHz, CDCl_3_): *δ* 5.75 (1H, s, H-4), 4.45–4.41 (1H, m, H-15), 1.23 (3H, s, H-19), 0.96 (3H, s, H-18).

Based on the regioselectivity and stereospecificity of the reaction, we hypothesize that the 15α-hydroxylation is catalyzed by a cytochrome P450 (CYP) monooxygenase system (Fig. S1). This is consistent with previous reports that filamentous fungi (*Gibberella* sp.) utilize CYP enzymes for steroid hydroxylation, as these enzymes exhibit high regio- and stereoselectivity for inert C–H bonds.^3,13^ We propose the following catalytic cycle for the stereoselective 15α-hydroxylation of ADD, step 1: substrate binding, step 2: oxygen activation, step 3: hydrogen abstraction at C-15, step 4: oxygen rebound and product formation.^13,29,30^ The stereoselectivity for α-hydroxy formation is governed by two key factors:Active site geometry, the CYP enzyme's active site is shaped to accommodate the steroid skeleton with the C-15α-face exposed to the ferryl-oxo species.^29^ Substrate orientation, ADD binds to the enzyme with its A/B rings in a specific conformation, aligning the C-15α-hydrogen with the reactive ferryl-oxo species. This orientation is stabilized by hydrogen bonding between the steroid's C-3 ketone group and a conserved arginine residue in the enzyme.^13,34^

### 3.4. Optimization of cultivation conditions for the production of 15α-OH-ADD by *Gibberella* sp. CICC 2498

**Fig. 5:**
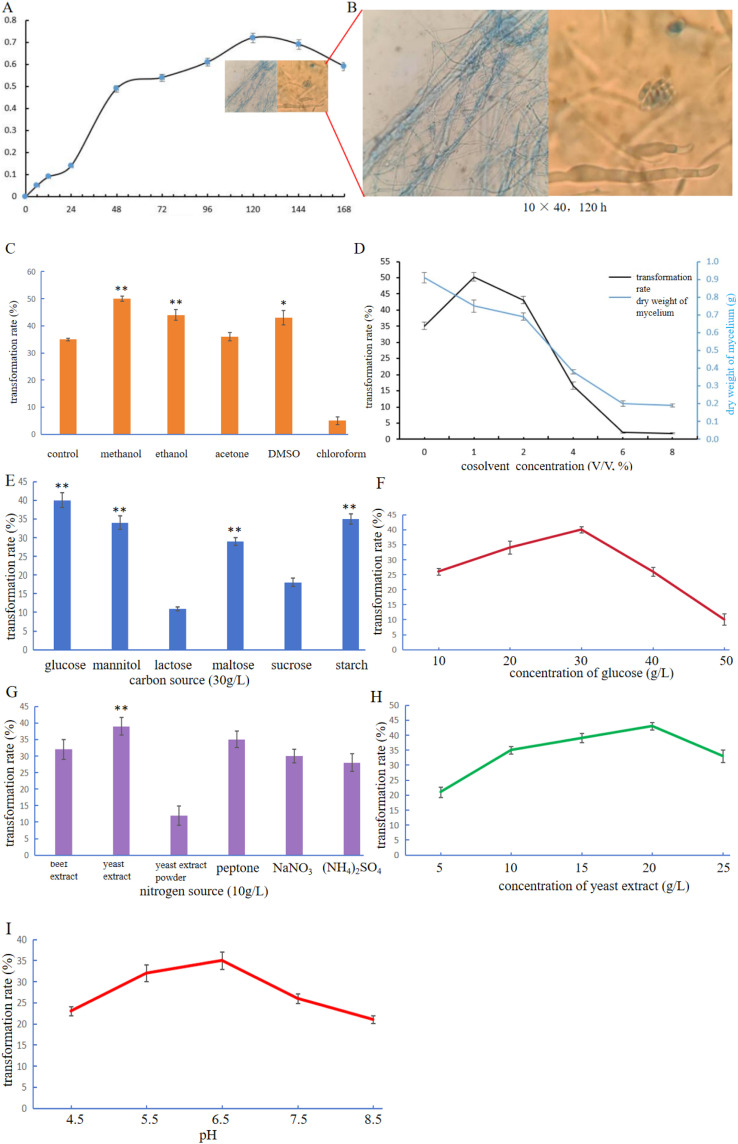
(A) Growth curve of *Gibberella* sp. CICC 2498. (B) Microscopic morphology of *Gibberella* sp. CICC 2498. (C) The influence of different cosolvents on the transformation rate. Conditions: 1 mL cosolvent (2%, V:V), ADD concentration 1 g L^−1^, pH 6.5, 28 °C, 220 rpm, 48 h. (D) The effect of varying methanol concentrations on the transformation rate. Conditions: ADD concentration 1 g L^−1^, pH 6.5, 28 °C, 220 rpm, 48 h. (E and F) The effect of different carbon sources and glucose concentration on the transformation rate. Conditions: ADD 1 g L^−1^, methanol 2%, yeast extract 10 g L^−1^, pH 6.5, 48 h. (G and H) The effect of different nitrogen sources and yeast extract concentration on the transformation rate. Conditions: ADD 1 g L^−1^, methanol 2%, sucrose 30 g L^−1^, pH 6.5, 48 h. (I) The effect of initial pH on the transformation rate. Conditions: ADD 1 g L^−1^, methanol 2%, yeast extract 10 g L^−1^, sucrose 30 g L^−1^, 48 h.

To optimize the biotransformation of ADD into 15α-OH-ADD by *Gibberella* sp. CICC 2498, growth characteristics of the fungus were first investigated. The growth curve of *Gibberella* sp. CICC 2498 was established by plotting dry cell weight against cultivation time, as shown in Fig. 5A. The fungal growth profile exhibited a clear lag phase from 0 to 24 h, followed by a exponential growth phase between 24 and 48 h, and a stationary phase from 48 to 144 h. After 144 h, the fungal culture entered a decline phase. Fig. 5B presents the morphological appearance of fungal mycelia at 120 h, demonstrating healthy growth and robust filamentous development suitable for biotransformation processes.

Subsequently, the effect of organic cosolvents on ADD biotransformation was systematically evaluated. The corresponding transformation yields at 48 h cultivation time are shown in Fig. 5C. Compared to the control group, the addition of methanol, ethanol, acetone, and DMSO significantly enhanced biotransformation yields. Among these solvents, methanol resulted in the highest biotransformation yield, and was thus selected as the optimal cosolvent for further experiments. The effects of different methanol concentrations on transformation yields are shown in Fig. 5D. The highest biotransformation rate was achieved at 1% methanol concentration. Lower concentrations of methanol were insufficient to fully dissolve the substrate, while higher concentrations exhibited toxic effects, adversely affecting fungal growth and hydroxylase enzyme activity, consequently reducing biotransformation efficiency.^31,34^

These optimized parameters establish an effective foundation for further enhancement and industrial scaling of the biotransformation process, promoting the sustainable and efficient production of steroid intermediates such as 15α-OH-ADD. Carbon sources play a crucial role in fungal growth, the biosynthesis of secondary metabolites, and the regulation of fungal signaling and mating pathways.^37,38^ As shown in Fig. 5E, glucose significantly outperformed all other tested carbon sources in promoting the transformation of ADD. Compared to the sucrose originally used in the basal medium, glucose led to a markedly higher transformation rate (Fig. 5F), and was therefore selected as the optimal carbon source for subsequent optimization studies. Nitrogen is an essential nutrient for fungal growth and is involved in the biosynthesis of proteins, amino acids, and a wide range of metabolic intermediates. The ability of fungi to metabolize diverse nitrogen sources enables them to thrive in various ecological niches and under nutrient-limited conditions.^39,40^ To determine the optimal nitrogen source for ADD biotransformation, both organic and inorganic nitrogen sources were tested (Fig. 5G). Among them, yeast extract yielded the highest transformation efficiency with a statistically significant difference compared to other groups. Notably, yeast extract was also the nitrogen source used in the original medium, further validating its suitability as the optimal nitrogen source. As shown in Fig. 5H, the transformation rate reached its peak at 20 g L^−1^, which was notably higher than the yield observed at the original concentration of 10 g L^−1^, thus identifying 20 g L^−1^ yeast extract as the optimal nitrogen concentration. Finally, the effect of initial pH on biotransformation efficiency was examined across a range of values. As shown in Fig. 5I, the transformation rate was highest at pH 6.5, which coincided with the initial pH used in the basal medium. This result highlights that maintaining a neutral to slightly acidic environment is favorable for maximizing the biotransformation of ADD by *Gibberella* sp. CICC 2498.

Based on the results of single-factor experiments, the optimal medium components were identified as follows: glucose as the preferred carbon source at a concentration of 30 g L^−1^, yeast extract as the optimal nitrogen source at 20 g L^−1^, and an initial medium pH of 6.5. Orthogonal test design and results are shown in Tables S1 and S2.^31,34^ According to the analysis of Table S2, the influence of the three factors on the transformation rate followed the order: initial pH (C) > carbon source concentration (A) > nitrogen source concentration (B). Range analysis (R) indicated that the optimal combination of factors was C2A1B1, corresponding to an initial pH of 6.5, glucose concentration of 20 g L^−1^, and yeast extract concentration of 15 g L^−1^. This optimal condition was not included among the original combinations tested in the orthogonal array, and thus required validation. To confirm the predicted optimal conditions, a validation experiment was conducted using three biological replicates under the optimal combination (glucose 20 g L^−1^, yeast extract 15 g L^−1^, pH 6.5). The average transformation rate reached 45.38%, representing a 2.87% improvement over the highest rate observed in the orthogonal test (42.51%) and a significant 15.03% increase compared to the original rate (30.35%). These results demonstrate that the orthogonal design and validation approach effectively enhanced the biotransformation efficiency of ADD to 15α-OH-ADD by *Gibberella* sp. CICC 2498, providing an optimized and statistically validated fermentation medium composition for potential scale-up and industrial application.

### 3.5. Optimization of biotransformation conditions for 15α-OH-ADD production by *Gibberella* sp. CICC 2498

**Fig. 6:**
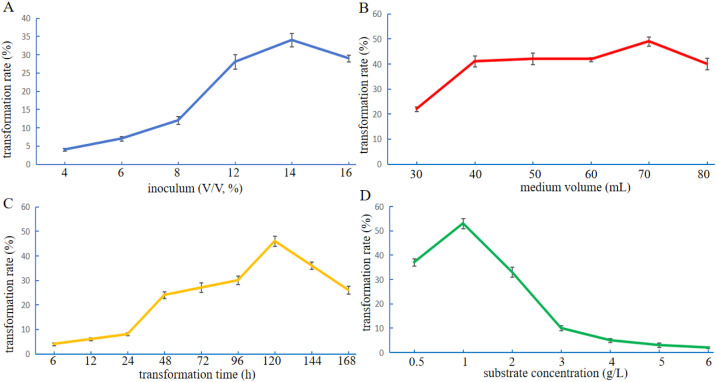
The effect of various transformation conditions on the production of 15α-OH-ADD by *Gibberella* sp. CICC 2498. The effect of inoculum size (A), medium volume (B), timing of substrate addition (C), and substrate concentration (D) on the transformation rate. Conditions: composition-optimized medium with initial pH 6.5, glucose 20 g L^−1^, and yeast extract 15 g L^−1^.

Optimization of biotransformation parameters is essential to maximize the production of 15α-hydroxyandrost-1,4-diene-3,17-dione (15α-OH-ADD) by *Gibberella* sp. CICC 2498. Several key process variables, including inoculum size,^41^ medium volume,^42^ transformation time,^43^ and substrate concentration,^44^ were evaluated to improve transformation efficiency. As shown in Fig. 6A, the highest biotransformation rate was achieved when the inoculum was 12%. Compared to the original condition (10%), a moderate increase in inoculum enhanced the frequency of contact between fungal biomass and substrate. However, excessive inoculum may reduce dissolved oxygen availability, thereby negatively affecting transformation rate.^45^ The effect of medium volume on transformation rate was assessed in 250 mL flasks (Fig. 6B). The optimal transformation rate was observed at a medium volume of 70 mL. A moderate increase in working volume improved the transformation rate, likely by maintaining adequate substrate availability and enzyme activity. Nevertheless, volumes exceeding 70 mL may reduce gas exchange, leading to lower oxygen transfer and suppressed transformation.^46^ The optimal substrate addition time was found to be at 120 h of cultivation (Fig. 6C). At this point, fungal biomass (dry weight) and hyphal integrity were at their peak, as evidenced in Fig. 5A and B. This suggests that hydroxylase accumulation was also at its maximum, providing favorable enzymatic conditions for efficient ADD transformation. As illustrated in Fig. 6D, the highest transformation rate was obtained when the substrate concentration was maintained at 1 g L^−1^. When substrate concentration exceeded 4 g L^−1^, biotransformation was nearly completely inhibited. This inhibition may be attributed to the increased concentration of organic cosolvent required to dissolve the higher substrate amounts. At elevated concentrations, these solvents can exert cytotoxic effects on fungal cells, suppressing both growth and hydroxylase activity,^47^ consistent with findings from Fig. 5D.

Collectively, these results underscore the importance of carefully balancing substrate availability, oxygen transfer, and solvent toxicity when optimizing the biocatalytic performance of *Gibberella* sp. CICC 2498. The identified optimal parameters provide a foundation for further scale-up and industrial application of this steroid biotransformation system. Based on the results of the single-factor experiments, the optimal biotransformation parameters were determined as follows: inoculum size of 12%, medium volume of 70 mL, transformation time of 120 h, and substrate concentration of 1 g L^−1^. To further refine the process conditions, statistical analysis of the experimental data was performed using SPSS 20.0. Factors and levels with statistically significant differences (*p* < 0.05) were selected for an orthogonal test design comprising four factors at three levels. The factors and their levels are summarized in Table S3, and the design and results of the orthogonal array are presented in Table S4. According to the results of Table S4, the order of influence of the four factors on transformation rate was: substrate concentration (A) > inoculum size (D) > medium volume (C) > transformation time (B). Range analysis (R) indicated that the optimal combination of conditions was A2D2C3B2, corresponding to a substrate concentration of 1 g L^−1^, inoculum size of 12%, medium volume of 80 mL, and transformation time of 120 h. As this specific combination was not included among the original experimental groups, a verification experiment was performed. The average transformation rate reached 61.74%, representing a 3.61% improvement over the highest rate (58.13%) observed in the orthogonal test and a remarkable 31.39% increase compared to the initial rate of 30.35%. These results confirm that the optimized biotransformation conditions significantly enhance the transformation rate of ADD to 15α-OH-ADD by *Gibberella* sp. CICC 2498. The successful implementation of a statistically guided optimization approach underscores the potential of this fungal strain for scalable, high-yield production of hydroxylated steroid derivatives for pharmaceutical applications. The introduction of the 15α-hydroxy group into the ADD molecule is not directly intended to enhance biological activity but rather to guide subsequent chemical reactions.

## 4. Conclusions

This study demonstrated the capability of whole-cell systems of *Gibberella* sp. CICC 2498 and *Absidia coerulea* CICC 41050 to biotransform androsta-1,4-diene-3,17-dione (ADD). The biotransformation by *A*. *coerulea* CICC 41050 resulted in multiple minor products with very low substrate conversion efficiency. In contrast, *Gibberella* sp. CICC 2498 produced two major transformation products, designated as product I and product II. Structural elucidation by HPLC, MS, and NMR confirmed that product I was 15α-hydroxy-androsta-1,4-diene-3,17-dione (15α-OH-ADD), and product II was 15α-hydroxy-androst-4-ene-3,17-dione (15α-OH-AD). The biotransformation process catalyzed by *Gibberella* sp. CICC 2498 was systematically optimized. Methanol at 1% (v/v) was identified as the most effective cosolvent. The optimal medium composition included glucose (20 g L^−1^) as the carbon source, yeast extract (15 g L^−1^) as the nitrogen source, and an initial pH of 6.5. Under these conditions, further optimization of process parameters yielded the following optimal transformation setup: substrate concentration of 1 g L^−1^, transformation time of 120 h, medium volume of 80 mL in 250 mL flasks, and inoculum of 12%. Under the optimized conditions, the transformation rate of 15α-OH-ADD reached 61.74%, representing a 31.39% improvement compared to the initial rate of 30.35%. These findings highlight the strong potential of *Gibberella* sp. CICC 2498 as a promising microbial biocatalyst for the regio- and stereoselective hydroxylation of steroid compounds and provide a solid foundation for the industrial-scale biosynthesis of pharmaceutically valuable hydroxylated steroids. Together, these findings lay a comprehensive foundation for the rational design of fermentation media to enhance steroid biotransformation efficiency in filamentous fungi.

## Author contributions

Ran He: methodology, investigation, writing – original draft. Ming Song: methodology, investigation, writing – original draft. Ruicheng Fu: writing – original draft. Yueying Jin: data curation. Shi Li: formal analysis, visualization. Fuju Wang: funding acquisition. Guogang Zhang: supervision. Yongbo Song: resources, methodology, supervision. Weizhuo Xu: resources, supervision, methodology, writing – review and editing.

## Conflicts of interest

There are no conflicts to declare.

## Supplementary Material

RA-016-D5RA08374E-s001

## Data Availability

All data supporting this study are available from the corresponding author upon reasonable request. The authors declare that all the data used for this manuscript can be found in its supplementary information (SI). Supplementary information is available. See DOI: https://doi.org/10.1039/d5ra08374e.
