# Supplementary material for: Stereoselective 15α-hydroxylation of androsta-1,4-diene-3,17-dione by Gibberella sp. for efficient production of 15α-OH-ADD and 15α-OH-AD
Source: RSC Adv. 2026 Jan 22;16(6):4766–75. doi: 10.1039/d5ra08374e (PMC12825004; doi:10.1039/d5ra08374e)
Supplement: RA-016-D5RA08374E-s001 [file RA-016-D5RA08374E-s001.pdf]

Supplementary Information (SI) for RSC Advances.
This journal is © The Royal Society of Chemistry 2025

Stereoselective 15α-Hydroxylation of Androsta-1,4-diene-3,17-dione by *Gibberella* sp. for Efficient Production of 15α-OH-ADD and 15α-OH-AD

Figure S1. Mass spectrum of product I (A) and product II (B).

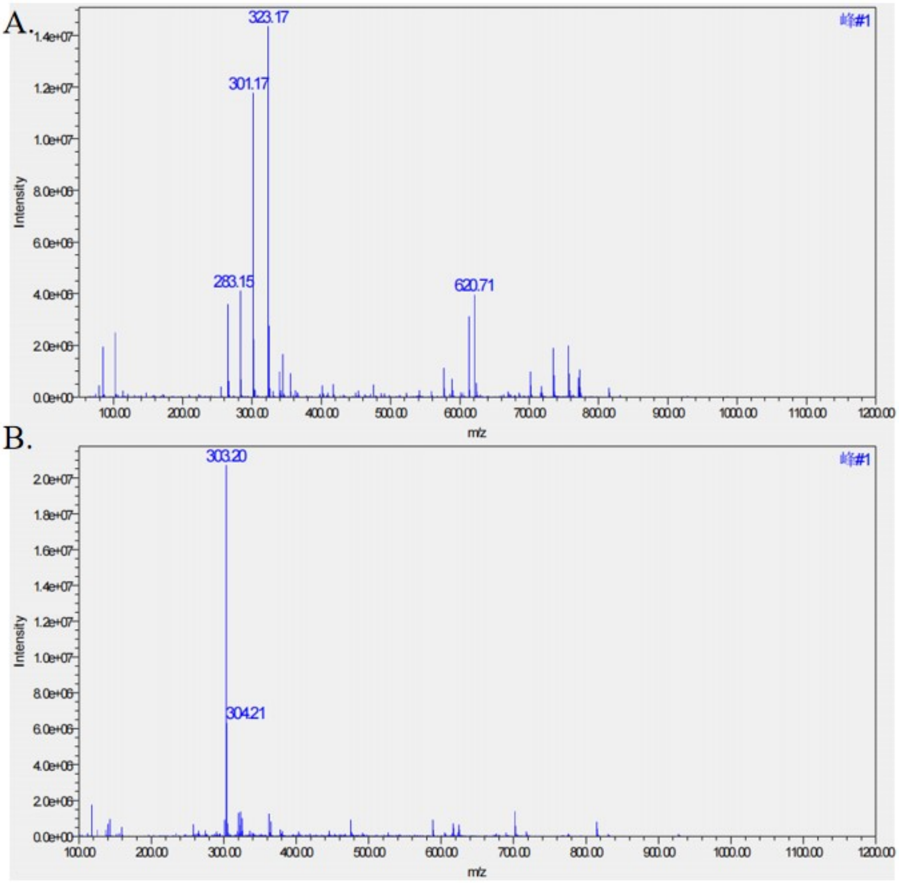

Figure S2. ^{13}C NMR of product I (A) and product II (B).

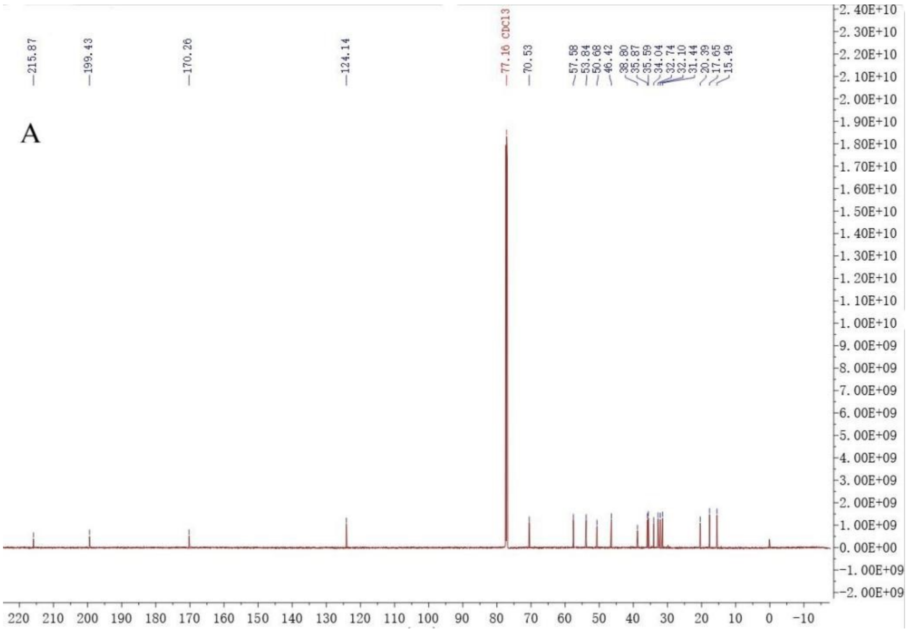

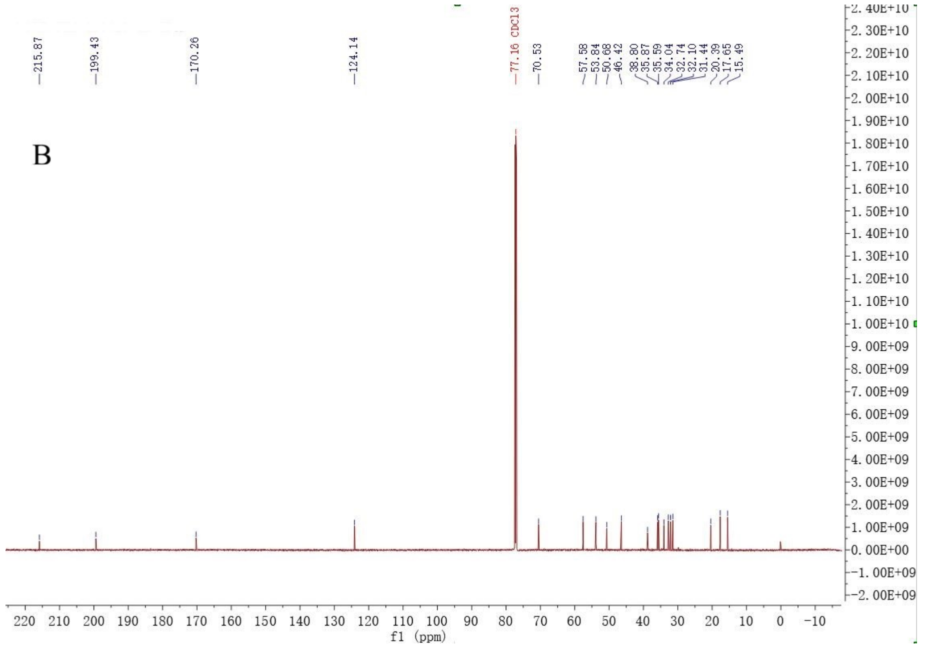

Figure S3. ^{1}H NMR of product I (A) and product II (B).

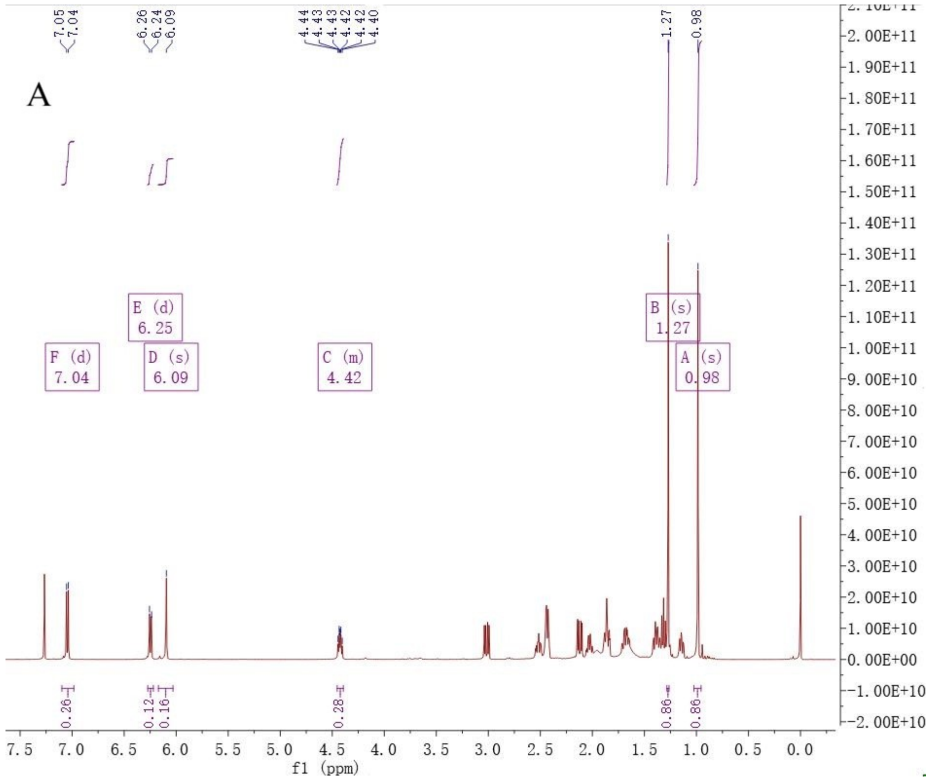

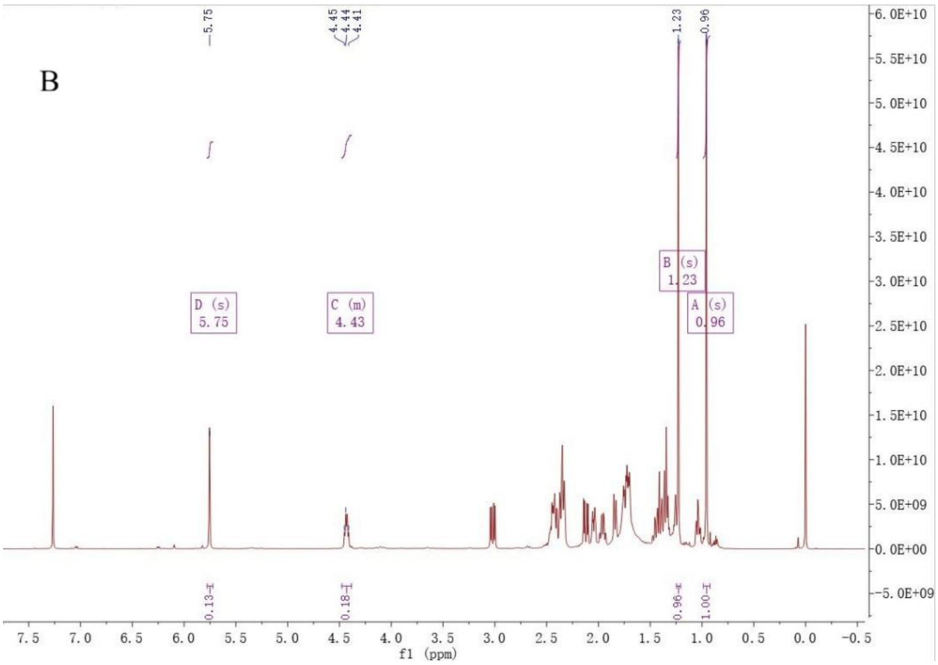

Table S1. Orthogonal experiment factors and levels

Factor / Level	A/glucose (g/L)	B/yeast extract (g/L)	C/initial pH
1	20	15	5.5
2	30	20	6.5
3	40	25	7.5

Table S2. Orthogonal experimental design and results

Factor / No.	A	B	C	Transformation rate (%)
1	1	1	1	40.74
2	1	2	2	39.89
3	1	3	3	37.56
4	2	1	2	40.72
5	2	2	3	21.02

6	2	3	1	26.22
7	3	1	3	26.48
8	3	2	1	36.21
9	3	3	2	42.51
K1	39.40	35.98	34.39	
K2	29.32	32.37	41.04	
K3	35.07	35.43	28.35	
R	10.08	3.61	12.69	

Table S3. Orthogonal experiment factors and levels

Factor / Level	A/ADD (g/L)	B/inoculum (V/V, %)	C/medium volume (mL)	D/time (h)
1	0.5	10	60	96
2	1	12	70	120
3	2	14	80	144

Table S4. Orthogonal experimental design and results

Factor / No.	A	B	C	D	Transformation rate (%)
1	1	1	1	1	44.38
2	1	2	2	2	50.93
3	1	3	3	3	45.61
4	2	1	2	3	44.21
5	2	2	3	1	58.13

6	2	3	1	2	49.92
7	3	1	3	2	46.48
8	3	2	1	3	37.12
9	3	3	2	1	36.67
K1	46.97	45.02	43.81	46.39	
K2	50.75	48.73	43.94	49.11	
K3	40.09	44.07	50.07	42.31	
R	10.66	4.66	6.27	6.80	10.66
